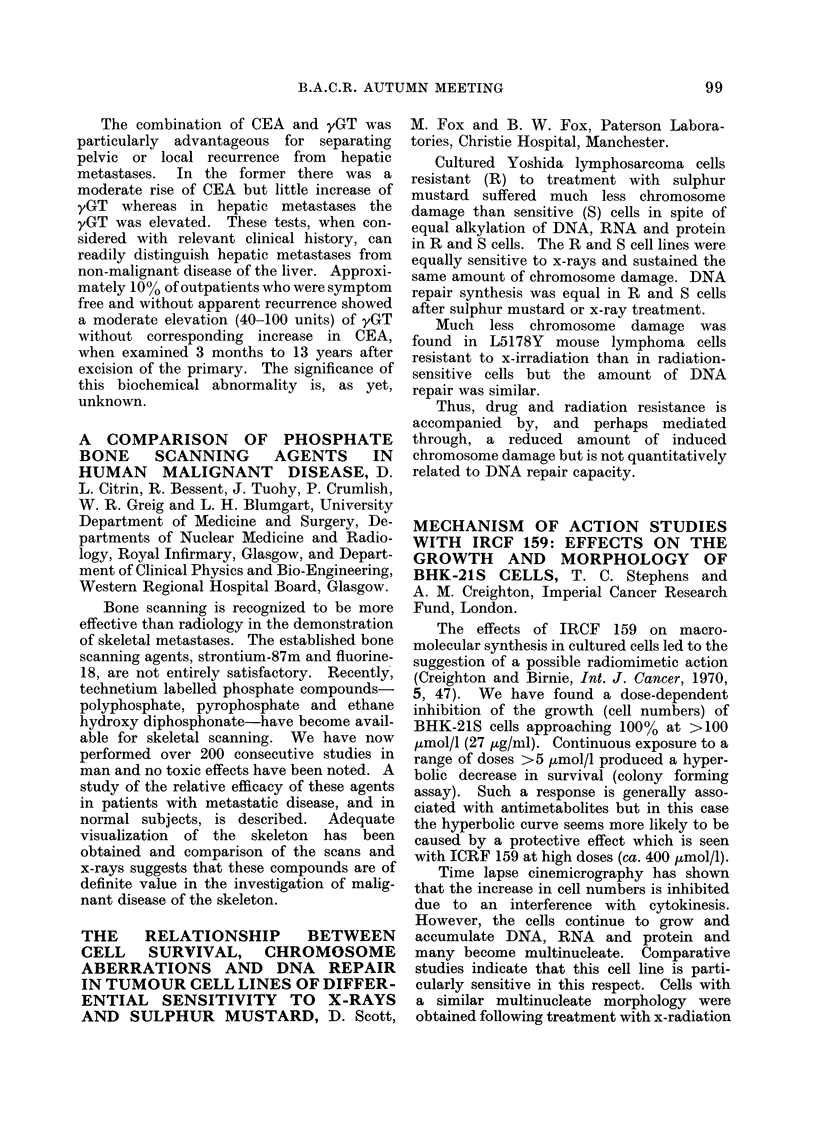# Proceedings: A comparison of phosphate bone scanning agents in human malignant disease.

**DOI:** 10.1038/bjc.1974.37

**Published:** 1974-01

**Authors:** D. L. Citrin, R. Bessent, J. Tuohy, P. Crumlish, W. R. Greig, L. H. Blumgart


**A COMPARISON OF PHOSPHATE BONE SCANNING AGENTS IN HUMAN MALIGNANT DISEASE,** D. L. Citrin, R. Bessent, J. Tuohy, P. Crumlish, W. R. Greig and L. H. Blumgart, University Department of Medicine and Surgery, Departments of Nuclear Medicine and Radiology, Royal Infirmary, Glasgow, and Department of Clinical Physics and Bio-Engineering, Western Regional Hospital Board, Glasgow.

Bone scanning is recognized to be more effective than radiology in the demonstration of skeletal metastases. The established bone scanning agents, strontium-87m and fluorine-18, are not entirely satisfactory. Recently, technetium labelled phosphate compounds—polyphosphate, pyrophosphate and ethane hydroxy diphosphonate—have become available for skeletal scanning. We have now performed over 200 consecutive studies in man and no toxic effects have been noted. A study of the relative efficacy of these agents in patients with metastatic disease, and in normal subjects, is described. Adequate visualization of the skeleton has been obtained and comparison of the scans and x-rays suggests that these compounds are of definite value in the investigation of malignant disease of the skeleton.